# Predictors of In-Hospital Mortality Following Pancreatectomy

**DOI:** 10.7759/cureus.45830

**Published:** 2023-09-23

**Authors:** Anna Axentiev, Artem Shmelev, Steven C Cunningham

**Affiliations:** 1 Surgery, Ascension Saint Agnes Hospital, Baltimore, USA; 2 Surgery, Columbia University, New York, USA

**Keywords:** predictors of mortality, post-operative mortality, postoperative pulmonary complications, pancreas disease, pancreas surgery

## Abstract

Background: In-hospital mortality rates following all types of pancreatic resections (PRs) have decreased over recent decades. Our aim was to identify predictors of in-hospital mortality following pancreatic resection.

Methods: All patients undergoing pancreatic resection were sampled from the National Inpatient Sample (NIS) in the years 2007-2012. Predictors of in-hospital mortality were identified and incorporated into a binary logistic regression model.

Results: A total of 111,568 patients underwent pancreatectomy. Annual mortality rates decreased from 4.3% in 2007 to 3.5% in 2012. Independent predictors of in-hospital mortality included age ≥75 years (vs. <65 years, OR = 2.04; 95% CI: 1.61-2.58), nonelective procedure status (OR = 1.46; 95% CI: 1.19-1.80), resection other than distal pancreatic resection (vs. Whipple, OR = 2.14; 95% CI: 1.71-2.69; other partial, OR = 2.48; 95% CI: 1.76-3.48), lower hospital volume (OR = 1.28; 95% CI: 1.09-1.49), indication for pancreatic resection other than benign diseases (vs. malignant, OR = 1.63; 95% CI: 1.25-2.15; other, OR = 2.48; 95% CI: 1.76-3.48), pulmonary complications (OR = 12.36; 95% CI: 10.11-15.17), infectious complications (OR = 2.17; 95% CI: 1.78-2.64), noninfectious wound complications and pancreatic leak (OR = 1.94; 95% CI: 1.53-2.46), and acute myocardial infarction (OR = 2.03; 95% CI: 1.32-3.06).

Discussion: Our findings identify predictors of inpatient mortality following pancreatectomy, with pulmonary complications representing the single most significant factor for increased mortality. These findings complement and expand on previously published data and, if applied to perioperative care, may enhance survival following pancreatectomy.

## Introduction

The first reports of pancreatic resection (PR) date back to the late 19th century [1], and multiple operations have since been described around the world. Although advances in pancreatic surgery techniques, including minimally invasive surgery, have drastically decreased mortality from as high as 30-50% historically to as low as <2% in contemporary surgery [1-13], postoperative morbidity and mortality remain substantial.

A combination of disease processes and patient-specific and hospital-level factors have been previously linked with increased morbidity and mortality following pancreatectomy [2-3,5-6,8-9,11]. However, there are not many recent studies at the national level, and a universally accepted approach to patient selection and perioperative management following PR is yet to be established. The aim of this study, therefore, was to identify predictors of in-hospital mortality following pancreatectomy using a national database.

## Materials and methods

Study population

The National Inpatient Sample (NIS) database, from 2007 to 2012, was queried for all admissions with ICD-9-CM (International Classification of Diseases, 9th edition, clinical modification) procedural codes for PR. These included proximal pancreatectomy, distal pancreatectomy, radical subtotal pancreatectomy, other partial pancreatectomy, total pancreatectomy, and radical pancreaticoduodenectomy (52.51, 52.52, 52.53, 52.59, 52.6, 52.7). With the intention of covering the entire cohort of patients undergoing PR, neither age cut-offs nor any other exclusion criteria were used. Our data captured patient demographics, PR type and indication, elective procedure status, hospital size, location, surgical volume, and teaching status. Weighting (provided in NIS for each record) was used to calculate nationwide estimates.

Statistical considerations

Potential predictors of in-hospital mortality were determined by the standard bivariate tests: the Chi-squared test, the independent-samples t-test, and the Mann-Whitney U test. We used an augmented list of codes for intra- and postoperative complications published by Simons et al. [14]. Accidental intraoperative injuries, included in this list, were captured according to our previously published method [15]. A multivariable logistic regression model was built using a forward stepwise approach, targeting the lowest possible Akaike information criterion. The pool of candidate predictor variables included patients’ sex, age group, race, Charlson comorbidity index, socioeconomic status, indication for PR (benign disease, malignancy, or other), type of PR (pancreatectomy [DP], Whipple pancreaticoduodenectomy [PD], total pancreatectomy), type of admission (elective or emergency), hospital PR volume tertile and teaching status, and complications (acute myocardial infarction, venous thromboembolism, gastrointestinal bleeding, respiratory, infectious and wound-related complications, pancreatic leak, and intraoperative accidental injuries). Multicollinearity was assessed by the variance inflation factor. NIS years 2000-2006 were additionally sampled to expand the study sample and add more longitudinal data for mortality trend analysis. Monthly mortality rates were calculated and analyzed with seasonal decomposition, isolating the trend and seasonal components of the mortality time series. All analyses were performed in R v. 3.5.1 (R Core Team 2018, Vienna, Austria).

## Results

Characteristics of the study population

A total of 111,568 (22,523 unweighted) patients underwent PR between 2007 and 2012. The study population consisted of an even ratio of male to female patients. Age was characterized by an unimodal distribution (60.3 ± 15.4), with individuals 55-74 years old constituting 51.8% of the entire study population. Patients ≥75 years old accounted for 17.6% of the study population.

**Figure 1:**
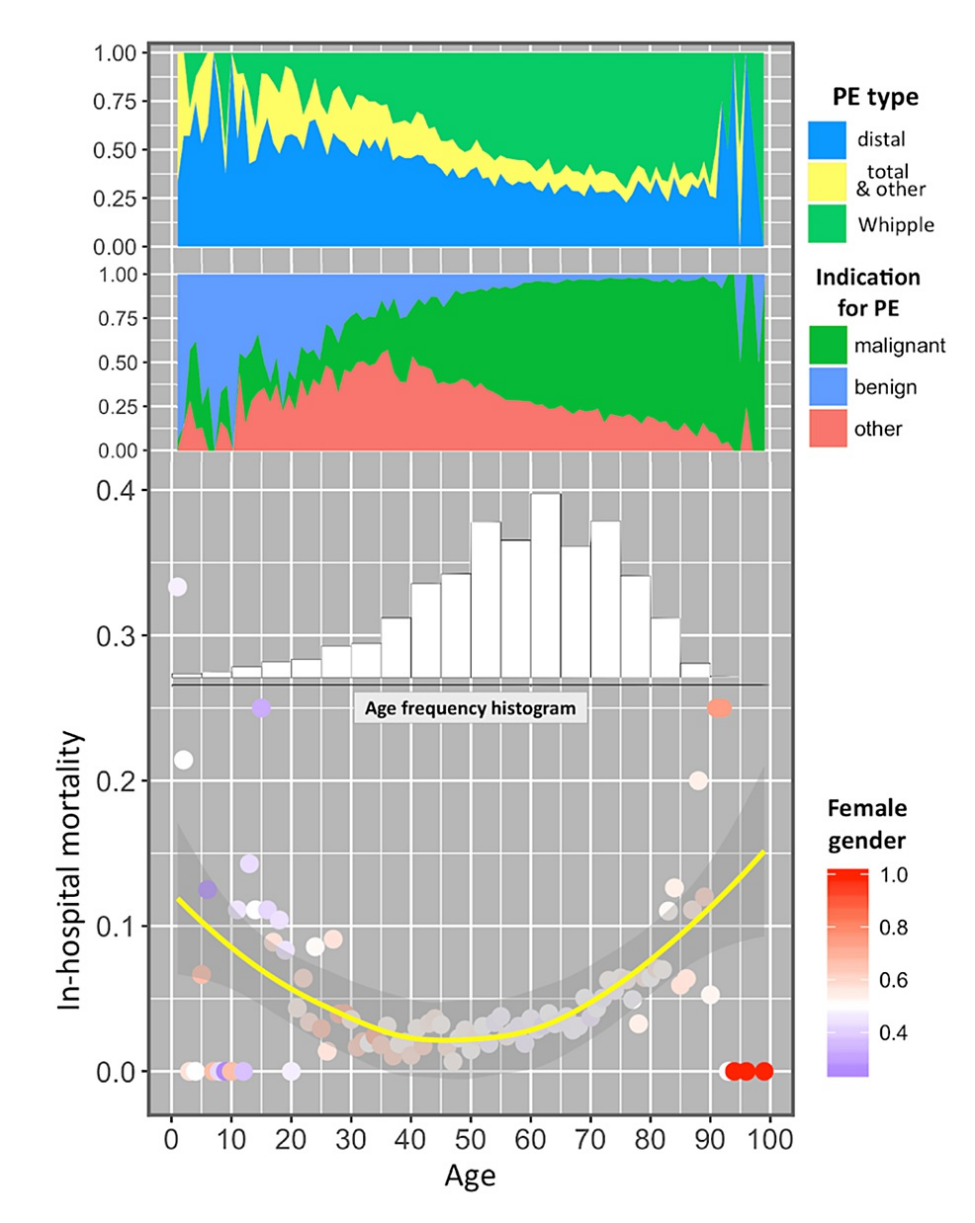
Structure of the study population, unweighted N = 22,523. Age-specific breakdown is provided by the type of pancreatic resection, indication for resection, gender, and mortality. The age-frequency histogram demonstrates contribution of each 5-year subgroup (e.g., individuals with age from 60 to 64) into the total size of study population. Proportion of the female gender within a given age is shown as a bicolor scheme, with red representing 100% females, blue – 100% males, and white – subsets with equal proportion of females and males (1:1). Mortality curve (yellow) is accompanied by a shaded area of 95% confidence intervals. Left axis demonstrates decimal fractions for each corresponding characteristic.

Overall, 79.3% of the patients underwent an elective pancreatic resection. Malignant indications accounted for 51.8% of all pancreatic resections (vs. benign disease, 28.7%, and others, 19.5%). Of all pancreatic resections, PD accounted for 55.3% (DP accounting for 33.9%, and other pancreatic resections for 10.8%). PR most commonly took place in teaching hospitals (83.1%), in an urban location (97.2%), and in hospitals with the highest pancreatic surgery volume (3rd tertile; 84.2%). The structure of the study population is provided in Figure 1. Younger individuals, predominantly males, most often underwent DP for benign diseases, while older patients more commonly had PD for malignant conditions.

In-hospital mortality trend and predictors

**Figure 2:**
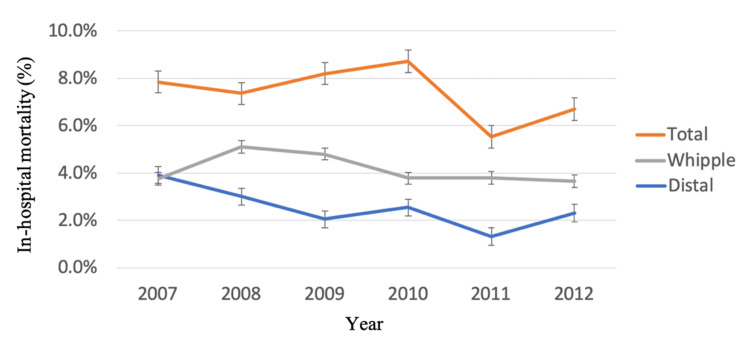
In-hospital mortality by pancreatic resection type for years 2007-2012.

Annual in-hospital mortality rates following pancreatic resection decreased from 4.3% in 2007 to 3.5% in 2012. This follows a decreasing mortality trend between the years 2000 and 2006, with respective mortality rates of 7.39% and 4.92%. In-hospital mortality by pancreatic resection type is displayed in Figure 2.

Predictors of in-hospital mortality following pancreatectomy are highlighted in Table 1. Age ≥75 years is shown to be an independent predictor of in-hospital mortality (vs. <65 years, OR = 2.04; 95% CI: 1.61-2.58). In-hospital mortality demonstrated a bimodal pattern with peaks towards extremes of age: 4.4% in those younger than 40 years old, 2.4% in 40-54 years old, 3.0% in 55-64 years old, 4.2% in 65-74 years old, and 6.7% in individuals 75 years and older. Independent predictors of in-hospital mortality included nonelective procedure status (OR = 1.46; 95% CI: 1.19-1.80), resection other than distal pancreatic resection (vs. Whipple, OR = 2.14; 95% CI: 1.71-2.69; other partial, OR 2.22; 95% CI: 1.65-2.97), lower hospital volume (OR = 1.28; 95% CI: 1.09-1.49), indication for pancreatic resection other than benign diseases (vs. malignant, OR = 1.63; 95% CI: 1.25-2.15; other, OR = 2.22; 95% CI: 1.65-2.97), as well as pulmonary complications (OR = 12.36; 95% CI: 10.11-15.17), infectious complications (OR = 2.17; 95% CI: 1.78-2.64), accidental puncture or laceration during surgery (OR = 2.01; 95% CI: 1.62-2.49), noninfectious wound complications and pancreatic leak (OR = 1.94; 95% CI: 1.53-2.46), and acute myocardial infarction (OR = 2.03; 95% CI: 1.32-3.06). Hospital size, location (urban vs. rural), and teaching status were collinear with pancreatic resections volume tertile (X2 p<0.001), so only volume was included in the final model.

**Table 1:** Logistic-regression model predicting in-hospital mortality following pancreatic resection. *Accidental intraoperative injuries detected by a composite marker.

Exponentiated regression coefficients	% CI:)	p-value
Age group (ref.: <65)		
65–74	1.63 (1.30–2.04)	<0.001
>75	2.04 (1.61–2.58)	<0.001
Indication for PE (ref.: benign condition)		
Malignant neoplasm	1.63 (1.25–2.15)	<0.001
Other	2.48 (1.76–3.48)	<0.001
PE type (ref.: distal)		
Whipple/proximal	2.14 (1.71–2.69)	<0.001
Total/other	2.22 (1.65–2.97)	<0.001
Surgical volume tertile (ordinal, 1–3)	0.78 (0.67–0.92)	0.002
Admission type (ref.: elective)		
Urgent/emergency	1.46 (1.19–1.80)	<0.001
Complications (ref: no complications)		
Pulmonary complications	12.36 (10.11–15.17)	<0.001
Infections	2.17 (1.78–2.64)	<0.001
Acute MI	2.03 (1.32–3.06)	0.001
Accidental puncture or laceration during surgery*	2.01 (1.62–2.49)	<0.001
Surgical wound complications	1.94 (1.53–2.46)	<0.001

## Discussion

Previous studies have reported mortality following PR ranging between 1% and 8% [2-10]. Our review of the NIS database revealed a decrease in mortality following pancreatectomy from 4.3% in 2007 to 3.5% in 2012, compared to in-hospital mortality of 7.3% in the year 2000. This is likely due to improved preoperative patient selection, operative technique, and postoperative management. It is imperative to scrutinize each aspect of perioperative care in order to improve outcomes following pancreatectomy.

Preoperative assessment of candidates for PR has been paramount in optimizing survival following surgery, but there is no one assessment tool universally standardized or accepted as the best. The Eastern Cooperative Oncology Group (ECOG) performance status scale has long been utilized to assess readiness to undergo major surgery [16]. Additionally, multiple validated scores have been proposed. For example, the WHipple-ABACUS score for 30-day mortality following PD takes into account hypertension, history of cardiac operations, age >62 years, bleeding disorder, albumin <3.5 g/dL, disseminated cancer, use of steroids, and preoperative systemic inflammatory response syndrome (SIRS) [8]. The PREPARE (Preoperative Pancreatic Resection) score for risk of major complications (Clavien-Dindo grades III-V) [17] following pancreatectomy includes systolic blood pressure, heart rate, hemoglobin level, albumin <3.5 g/dL, ASA class >II, surgical procedure, elective surgery status, and disease of pancreatic origin [7]. Indeed, the Clavien-Dindo classification system for complications has been applied to and validated for the grading of complications following pancreatic surgery in particular [18]. The SOAR score for risk of in-hospital mortality following PR includes age, Charlson score, sex, preoperative diagnosis (malignant status), type of resection, and hospital volume [5]. The American College of Surgeons National Surgical Quality Improvement Program (ACS-NSQIP) ‘Pancreatectomy Risk Calculator’ includes age >74 years, male gender, BMI >40, preoperative sepsis, functional status, ASA class >II, history of coronary heart disease, dyspnea on exertion, bleeding disorder, and extent of surgery [3]. Such risk-assessment tools take into account factors most commonly associated with morbidity and mortality following PR but should be used with caution as some studies have suggested that they may underestimate mortality events [12]. Our study additionally demonstrates that patients at extremes of age (<34 years and >75 years) who undergo nonelective pancreatic resection in the setting of malignancy or traumatic injury at low-volume centers are at higher risk of in-hospital mortality. Preoperative identification of high-risk patients is an important avenue for improving survival after PR.

Given the importance of technique and the inherent risk of injury in pancreatic surgery, we examined accidental intraoperative injuries, showing that such injuries were associated with doubled in-hospital mortality. These data do not reveal if such injuries were due to challenging dissection attributable to locally advanced disease leading to unavoidable violation of adjacent viscera or requiring vascular reconstructions, due to other patient-related disease, or to other factors. Surgeons attributed technical errors or poor intraoperative judgment to poor outcomes in 21% of the cases, as per the root-cause analysis of >10,000 pancreatectomies [12]. Besides technical errors, other intraoperative factors are associated with increased morbidity and mortality after pancreatic resections, likely representing the complexity of the case. These include increased operative time and blood loss, a high intraoperative transfusion requirement, vascular repair, and multivisceral resections [10-12]. Ongoing advances in operative technique and perioperative care led to a substantial decrease in mortality following PR in specialist hands. This can be optimized further with the application of minimally invasive surgical techniques. Laparoscopic and robotic techniques have been associated with decreased intraoperative blood loss and improved postoperative outcomes in pancreatic surgery [19-20].

The overall decrease in mortality following PR has been attributed in part to the implementation of a multidisciplinary approach to postoperative patient care, including the utilization of interventional radiology and intensive care unit optimization in high-volume hospitals. Complications such as postpancreatectomy hemorrhage and frequently coinciding pancreatic fistulas have been implicated in mortality following pancreatectomy [10]. Additionally, cardiac arrest, postoperative shock, and acute kidney failure have all been associated with worse outcomes [9,12]. Those may be noticed and addressed more promptly in units of higher acuity, as seen by lower failure-to-rescue rates in high-volume centers [21].

In this study, we demonstrate that the presence of pulmonary complications is by far one of the most significant predictors of mortality following pancreatectomy. Pulmonary complications encompass aspiration pneumonitis, respiratory failure, and the need for mechanical ventilation. Prior studies have reported on this correlation with pneumonia and respiratory distress, which was shown to be more prevalent in those patients who experienced mortality following pancreatectomy [12]. Pneumonia and ventilator dependence lead to significant complications (Clavien-Dindo grades IV-V) 26.5% of the time and an increased risk of mortality within 90 days of pancreatic surgery with an odds ratio of 9.6 [22-23]. Nagle et al. identified delayed gastric emptying, supplemental oxygen requirements on postoperative day 3, and the presence of chronic obstructive pulmonary disease as independent risk factors for the development of pneumonia following pancreatectomy and the associated morbidity and mortality [23]. Our study further supports the correlation between pulmonary status and outcome following PR. Optimizing preoperative respiratory status and implementing strict pulmonary toileting practices in the postoperative setting may therefore improve survival following PR.

Our study has limitations that are inherent in administrative databases, including the difficulty of assessing the severity of comorbid diseases and distinguishing pre-existing comorbidities from postoperative ones. Within the limitations of this kind of study, we highlight predictors of in-hospital mortality following pancreatectomy using a large, national database. Additionally, although our query of the NIS database is limited to the years 2007 and 2012 and thus utilizes exclusively ICD-9 codes, our results remain relevant to present-day practice. Our literature review was maintained current and reveals that our results add to and strengthen the wealth of data highlighting risk factors for in-hospital mortality after PR.

## Conclusions

In summary, our findings identify predictors of inpatient mortality following PR, with pulmonary complications representing the single most significant factor for increased mortality, followed by other modifiable and non-modifiable factors including increasing age, malignant disease, type of pancreatic resection, intraoperative injury, postoperative infection, and myocardial infarction. This complements and expands on previously published data. Although mortality continues to decline following PR, our study suggests an important avenue for further improving outcomes following pancreatic surgery, namely respiratory prehabilitation. Ongoing focus on preoperative patient selection and optimization, operative decision-making, and postoperative care can further enhance survival following PR.
